# Detection of Porcine Norovirus GII.18 Strains in Pigs Using Broadly Reactive RT-qPCR Assay for Genogroup II Noroviruses

**DOI:** 10.1007/s12560-024-09619-x

**Published:** 2024-12-30

**Authors:** Ankita K. Gupta, Mari Heinonen, Emilia König, Venla Mikkonen, Leena Maunula

**Affiliations:** 1 Department of Food Hygiene and Environmental Health, Faculty of Veterinary Medicine, University of Helsinki, Helsinki, Finland; 2 Department of Production Animal Medicine, Faculty of Veterinary Medicine, University of Helsinki, Helsinki, Finland

**Keywords:** Caliciviridae, Human norovirus, Porcine norovirus, RT-qPCR, Genotyping

## Abstract

Noroviruses, belonging to the family *Caliciviridae*, are classified into at least ten genogroups (G) based on their major capsid protein (VP1). The common genogroup to be identified in both humans and pigs is GII, although porcine noroviruses (PoNoVs) belong to genotypes of their own (GII.11, GII.18, and GII.19). So far, PoNoVs have not been studied much in Finland, possibly due to their rather symptomless nature in pigs. In the present study, we enrolled a total of 189 fecal samples collected from pigs from Finnish farms. Samples were taken from 12 farms in 2010, 2019 and 2020. We analyzed feces from growing pigs ranging from 2.1 to 6 months of age. RNA was extracted from fecal suspensions using a commercial viral RNA extraction kit, followed by RT (reverse transcription)-qPCR. The genotypes were determined by Sanger sequencing of the PCR fragments amplified by conventional PCR. Of the 12 farms, 6 (50%) had at least one PoNoV-infected pig. Altogether 18 (9.5%) of the 189 pigs tested positive for PoNoVs. Pigs mostly aged over 4 months were infected with PoNoVs. Eventually, 12 positive samples were determined as genotype GII.18. We could demonstrate the presence of PoNoVs in Finnish pigs. In future, more studies in which longer sequences from PoNoV genome can be obtained, are required.

## Introduction

Noroviruses belong to the *Caliciviridae* family. *Norovirus* genus has a single representative species named Norwalk virus (Lefkowitz et al., 2018). Among foodborne viruses, norovirus is one of the leading pathogens, causing epidemic gastroenteritis in humans worldwide (Cavicchio et al., 2022; Robilotti et al., 2015). In humans, noroviruses cause a disease with symptoms of vomiting, diarrhea, abdominal pain, and often fever. The virus is usually spread by the fecal–oral route. This may be through contaminated food or water or person-to-person contact. It may also spread via contaminated surfaces or through air from the vomit of an infected person.

NoVs are a genetically diverse group with a single-stranded RNA genome of approximately 7.5–7.7 kilobases and icosahedral structure. The genome of most noroviruses is organized into three open reading frames (ORFs), except for murine noroviruses, which contain a fourth ORF (Chhabra et al., 2019). The genomic structure of norovirus consists of polyprotein genes that produce several nonstructural proteins, including an RNA-dependent RNA polymerase (RdRp), and then of a major capsid protein gene (VP1), and a minor capsid protein gene (VP2) (Wang et al., 2005).

Noroviruses are classified into ten genogroups based on genetic variation of the major capsid protein (VP1) gene and are further divided into 49 genotypes (Chhabra et al., 2019). The genogroup GI (GI.1–GI.9) and GII (GII.1–GII.10, GII.12–GII.17 and GII.20–GII.27) noroviruses infect humans (Parra, 2019). Particularly GII.4 causes up to 50% of human gastroenteritis epidemics worldwide (Kendra et al., 2022). PoNoVs also belong to genogroup GII, genotype GII.11, GII.18, and GII.19 (Silva et al., 2015). In 1998, norovirus RNA was first detected in adult porcine fecal samples in Japan and then in Europe and the USA, as reviewed by van der Poel et al. (2000).

Thus, porcine norovirus and some human norovirus genotypes belong to the same GII genogroup. However, no porcine noroviruses have been found to infect humans, whereas studies have found that some human noroviruses can infect pigs. Human norovirus GII.4 infecting pigs was reported in Canada in 2005–2007 (L’Homme et al., 2009; Mattison et al., 2007), then GII.3, GII.4, and GII.13 in Japan in 2008–2009 (Shen et al., 2012), GII.1 in Ethiopia in 2013 (Sisay et al., 2016) and GII.2 in Dutch–German border region in 2017–2018 (Schuele et al., 2021).

In norovirus genome, recombination breakpoint often locates between the capsid gene and a polymerase gene (RdRp), a junction region (Kendra et al., 2022). Recombinant porcine noroviruses have also been identified in pigs (Shen et al., 2012). Shen et al. (2012) found that the Chinese recombinant strain pNoVs (Ch6) was distinct from the known porcine GII strains (GII.11, GII.18, and GII.19), possibly a novel genotype, based on capsid region, whereas the RdRp region was grouped into the GII.11 cluster. Wang et al. (2005) have suggested that GII.18 could be a recombinant between human and porcine norovirus, since it is genetically more distant from GII.11 and GII.19 (Wang et al., 2005). In an experimental study, it was seen that gnotobiotic miniature pigs infected with this recombinant virus showed symptoms, like mild diarrhea (Shen et al., 2012), but in general, no symptoms are seen in pigs infected with PoNoVs.

There may be underexplored routes of transmission, including indirect zoonotic transmission, where an animal or environmental reservoir plays a role in spreading the virus to humans through food products. A study done in Canada tested fecal samples obtained from pig and dairy farms, as well as retail meat samples for the norovirus genome. They found a GII.4-like strain of norovirus associated with a retail raw pork sample (Mattison et al., 2007). Shellfish concentrate noroviruses efficiently from growing waters and viruses can be spread to humans, if for example contaminated oysters are eaten uncooked or very mildly cooked. Norovirus outbreaks linked to shellfish consumption are continuously being reported around the world (Gyawali et al., 2019).

After the first detection of PoNoVs in Japan (Sugieda et al., 1998), there have been reports of PoNoVs of different genotypes on six continents so far: Asia, Europe, Africa, North and South America, and Oceania as reviewed (Cavicchio et al., 2022). In the literature, the percentage of positive PoNoVs ranges from 9% to over 50% (Cavicchio et al., 2020; Nakamura et al., 2010; Silva et al., 2015; Wang et al., 2006; Wolf et al., 2009).

Reverse Transcription (RT)-PCR is currently the most popular method for norovirus detection in both clinical and food samples (Atmar & Estes, 2001; Gyawali et al., 2019; Mattison et al., 2011). PoNoV is closely related to human norovirus, and there is evidence that broad-range RT-PCR assays targeting HuNoV also detect PoNoVs (L’Homme et al., 2009).

PoNoVs have not been much studied in Finland. The aim of this study was to evaluate the presence of PoNoV in Finnish pig herds. In this study, we used real-time RT-qPCR test with primers designed to detect a large variety of GII norovirus. Our study also reveals the partial genomic sequences of PoNoV strains.

## Materials and Methods

### Sampling

**Fig. 1:**
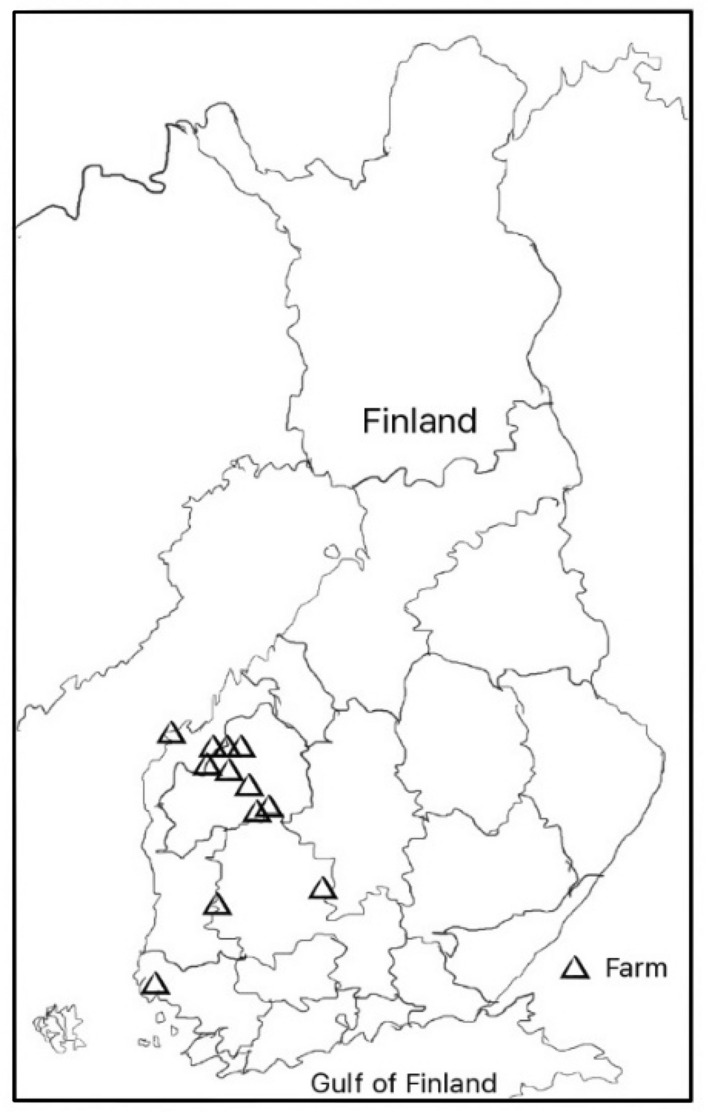
Location of 12 Finnish farms sampled for the presence of porcine noroviruses

Porcine fecal sample material (n = 189) for this study was selected from two study materials collected for other projects: a study linked to investigation of virus transmission among pigs during 2010 (Kantala et al., 2013) and a study linked to investigation of fecal microbiota in asymptomatic pigs on 11 farms between February 2019 and March 2020 in Finland. The geographic distribution of these volunteer farms is illustrated in Fig. 1. The herds had an average of 1953 finishing pigs (standard deviation, SD 1345 pigs, 12 herds) and 800 sows (SD 424, 2 herds). Samples were collected from weaned and finishing pigs with disposable gloves directly from the rectum of individual pigs and transferred into plastic tubes immediately after collection. After sampling, the tubes were kept in a cool box, moved to − 18 °C within one hour and to − 70 °C on the following day at the latest, where they were kept until the start of laboratory analyses.

### Preparation of Fecal Suspensions

After thawing the samples, 10–20% (w/v) fecal suspensions were prepared by adding fecal materials to a phosphate buffered saline (PBS) solution. These fecal suspensions and the archived samples were stored frozen at − 20 °C or at − 70 °C at the Department of Food Hygiene and Environmental Health, University of Helsinki, Finland.

### RNA Extraction

RNA was extracted from the 10–20% fecal suspensions prepared in PBS solution. Briefly, 150 μl of suspension was taken for RNA extraction by using EZNA® Viral kit (Omega Bio-Tek, Germany), according to the manufacturer’s instructions. The RNA was eluted in 50 µl of RNase-free water and stored at − 20 °C. Mengovirus (from A. Bosch, University of Barcelona, Spain) was used as a process control and murine norovirus MNV-1 as a control for PCR inhibition. As a negative control, PBS was used.

### RT-qPCR

We used the commercial QuantiTect® Probe RT-PCR kit (Qiagen) for primary detection of PoNoV. It includes the enzyme Reverse Transcriptase and Master Mix solution with nucleotides, polymerase enzymes, and a buffer for PCR amplification. Forward and reverse primers and a probe solution were added to a final concentration of 1 µM each and 0.2 μM, respectively (Table 1). To every 15 μl of Master Mix, 5 μl of sample was added, and the final volume prepared was 20 μl to run the RT-PCR. All samples were done in duplicate.

**Table 1:** Oligonucleotides used to study porcine noroviruses in pigs

Primers used for RT-qPCR	Sequence (*)	Source
Norovirus GII (NoV)		
QNIF2 (FW)	5′ATGTTCAGRTGGATGAGRTTCTCWGA-3′	Loisy et al. (2005)
COG2R (RV)	5′-TCGACG CCATCTTCATTCACA-3′	Kageyama et al. (2003)
QNIFs (PROBE)	FAM-AGCACGTGGGAGGGCGATCG-BHQ1	Loisy et al. (2005)
Mengo virus (MeV)		
Mengo 110 (FW)	5′-GCGGGTCCTGCCGAAAGT-3′	Pintó et al. (2009)
Mengo 209 (RV)	5′-GAAGTAACATATAGACAGACGCACAC-3′	
Mengo 147 (PROBE)	FAM-ATCACATTACTGGCCGAAGC-BHQ1	
Murine norovirus (MNV)		
MNV (FW)	5′-TGCAAGCTCTACAACGAAGG-3′	Hewitt et al. (2009)
MNV (RV)	5’-CACAGAGGCCAATTGGTAAA-3’	
MNV (PROBE)	FAM-CCTTCCCGACCGATGGCATC-BHQ1	
Primers used for genotyping		
G2SKF (FW)	5′-CNTGGGAGGGCGATCGCAA-3′	Jiang et al. (1999)
G2SKR (RV)	5′-CCRCCNGCATRHCCRTTRTACAT-3′	
p290 (FW)	5′-GATTACTCCAAGTGGGACTCCAC-3′	Kojima et al. (2002)
p289 (RV)	5′-TGACAATATAATCATCACCATA-3′	
GII.18 FoP (FW)	5′-GTTGATGCTTTGAGYGCGCCC-3′	This study
GII.18 RoP (RV)	5′-GGCTTGCTTTTGGACTCAACTGT-3′	
GII.18 FiP (FW)	5′-ACAATGTGCCCCCACGTTATAG-3′	
GII.18 RiP (RV)	5′-GGTGGTATCAAAAAGGTGAACTC-3′	

*Genome locations: G2SKF (5058–5076), G2SKR (5379–5401), p290 (4568–4590), p289 (4865–4886), GII.18 FoP (5451–5471), RoP (5720–5738), FiP (5478–5490), and Rip (5696–5718)

RT-qPCR reactions were carried out using the Rotor-Gene 3000 cycler instrument (Corbett Research, Qiagen GmbH). RT reaction for 25 min at 50 °C started each run and was followed by preheating at 95 °C for 15 min. The 45 amplification cycles consisted of a denaturation step at 95 °C for 15 s and an annealing-extension step at 60 °C for 60 s. A positive control comprising a human norovirus GII strain (GII.4, anonymous) and a negative control of RNase/DNase free water were used.

### PEG Precipitation

The fecal suspension positive for PoNoV underwent RNA extraction with or without concentration treatment using polyethylene glycol (PEG). The treatment was performed according to Summa et al. (2012), with some modifications. The success of PEG precipitation was confirmed by demonstration of a decrease of the Ct values obtained by RT-qPCR after the experiment. Fecal suspensions of 5 ml were added to 25 ml of 100 mM Tris-50 mM Glycine 1% Beef Extract (TGBE) Buffer. This mixture was agitated for 20 min, 150 rpm on a shaker (IKA) at room temperature, followed by centrifugation for 30 min, 10,000×g, 8 °C. The supernatant was transferred to another tube quickly after centrifugation had finished. The pH of the supernatant was adjusted to 7–7.5 by adding HCl. A solution of 5 × PEG/NaCl (500 g/l PEG 8 000, 1.5 M NaCl) was added, and the mixture was agitated at 6 °C, 250 rpm for 1 h on a shaker (IKA). Then, the samples were centrifuged for 30 min, 10,000×g, 8 °C. The supernatant was removed, and the pellet was eluted with 300 μL of PBS. Samples were stored at − 70 °C or RNA was directly extracted by using EZNA® Viral kit.

### Conventional RT-PCR, Nested PCR, and Sanger Sequencing

A selection of samples detected positive by RT-qPCR were first amplified by conventional PCR assays (OneStep RT-PCR Kit, QIAGEN®) using primer pairs G2SKF/G2SKR or p289/290 (Table 1). Each reaction included 5 μL of 5X Qiagen One-Step RT-PCR Buffer, 1 μL of dNTP mix, and 1 μL of Qiagen One-Step Enzyme mix (these reagents are included in the kit). Each primer at a final concentration of 0.6 μM (Table 1), 0.15 μL of RNAse inhibitor, and 11.85 μL of RNAse-free water were added to the mixture. Positive controls of human norovirus (GII.4, anonymous) and PoNoV (322-2, kindly sequenced earlier by Dr. Kantala, now confirmed as GII.P11; GenBank accession no. PP479753) were included in all experiments, as were negative controls. Conditions of PCRs were as follows: the samples were first incubated at 42 °C for 55 min and then the initial denaturation was performed at 95 °C for 15 min; 39 cycles were performed. Each cycle consisted of denaturation at 94 °C for 20 s, primer annealing at 51 °C for primer G2SKF/G2SKR and 60 °C for p289/290 for 30 s, and an extension step at 68 °C for 1 min, followed by a final extension at 68 °C for 10 min.

All positive samples were also amplified with nested RT-PCR using primers designed by us for GII.18 (Table 1), as the tests with primer pairs G2SKF/R and p290/289 were mostly unsuccessful. PCR-1 consisted of a 30-min initial incubation at 55 °C, followed by a 15-min enzyme activation at 95 °C, then a 1-min denaturation at 94 °C, 1-min annealing at 58 °C, and 1-min extension 72 °C, all three steps repeated 30 times, followed by a 10-min extension at 72 °C. In PCR-2, the initial incubation step was deleted, and the program was set for 39 cycles with the same conditions as PCR-1. All reactions were performed in a thermal PCR cycler (BIO-RAD T100).

The PCR products were run in 2.5% agarose gel (Seakem, Rockland, ME USA), at 105 V, 300 A for 60 min. GeneRuler™ DNA Ladder (Thermo Fisher Scientific™) was used as a molecular marker. Each gel was visualized with SYBR™ Safe DNA Gel Stain (Thermo Fisher Scientific) with blue light filter. PCR products of a correct length (330 bp for G2SKF/R, and 320 bp for p289/290, 240 bp for nested PCR) underwent Sanger sequencing (DNA Sequencing and Genomics Laboratory, BIDGEN).

### Sequence Analysis

Nucleic acid sequences of the PoNoV partial capsid gene (regions 5478–5718 and 5058–5076 based on sequence AY823305) obtained were compared with those available in Genbank using BLAST (https://blast.ncbi.nlm.nih.gov/Blast.cgi). The sequences were aligned with the Unipro UGENE software version 49.1 (http://ugene.net/). Phylogenetic analysis was performed using the norovirus genotyping tool (https://www.rivm.nl/mpf/typingtool/norovirus/) and EMBL phylogenetic tool (https://www.ebi.ac.uk/jdispatcher/msa). The sequences obtained in this study were deposited into GenBank (accession no: 3627-8n-Fi: PP475402, 2908-6n-Fi: PP475403, 3622skf-Fi: PP479669, and 3622-1n-Fi: PP479670).

## Results and Discussion

### Detection of PoNoVs in Pigs

In this study, we investigated the presence of PoNoVs in Finnish pigs by the RT-qPCR method, which is also used for the detection of human GII NoVs (L’Homme et al., 2009). We used norovirus primers and a probe targeting the viral junction of RdRp and capsid gene (primers QNIF2 and COG2R, and probe QNIFs), and we were able to identify PoNoVs in porcine feces.

We tested a total of 189 porcine fecal samples, 18 (9.5%) of which were positive for PoNoV by GII RT-qPCR assay (Table 2). We found at least one positive sample in half of the 12 farms. The highest percentage of samples positive for PoNoV on one farm was 41.2%. Detailed farm-specific results are presented in Table 2.

**Table 2:** Number and percentage of fecal samples positive for porcine noroviruses (PoNoVs) in 12 Finnish pig farms

	Farm number	Number of PoNoV positive samples/total	% of positive samples
Farms	3	0/3	0
	4	0/4	0
	5	0/3	0
	6	0/17	0
	7	0/13	0
	**8**	**3/18**	**16.7**
	**9**	**2/18**	**11.1**
	10	0/26	0
	**11**	**1/19**	**5.3**
	**12**	**7/17**	**41.2**
	**13**	**1/19**	**5.3**
	**15**	**4/32**	**12.5**
Total		18/189	**9.5**

Positive farms are indicated in bold

In our study, the overall percentage of positive PoNoVs of 9.5% based on the RT-qPCR method was in accordance with a previous study using a semi-nested PCR method that was conducted in New Zealand, where the percentage of positive PoNoVs was 9% (Wolf et al., 2009). A Brazilian study reported a higher number, 51.18% (Silva et al., 2015), and many other countries found a somewhat lower percentage of positive samples: 16.6% in Japan (Nakamura et al., 2010), 11.4% in Northeast Italy (Cavicchio et al., 2020), 18.9% in North Carolina, USA (Scheuer et al., 2013), and 20% in the United States overall (Wang et al., 2006). We did not have enough samples and farms to conduct a proper prevalence study. Differences in the percentage of positive samples in other studies could be due to different sampling strategies, geographical locations and climate, ages of pigs sampled, or detection methods used, for instance, different primers with various specificities and running conditions in PCR for PoNoVs.

As mentioned in the introduction, some primers that recognize GII HuNoVs also attach to the PoNoV genome since the attachment sites for the primers and probe have a high nucleic acid identity level (L’Homme et al., 2009). Our results supported this finding and demonstrated that at least GII.18 PoNoV strains are detected with the primers commonly used in the GII human norovirus RT-qPCR assay. The stringency level, such as the RT or annealing temperature, set to the assay was found to affect the number of the porcine fecal samples yielding positive results (data not shown). Temperatures of 50 °C for the RT step and 60 °C for the combined annealing and extension step were used in this study to detect PoNoVs, although more stringent PCR running conditions are generally used for human norovirus. Our study also explains our earlier observation in which GII NoVs were detected in porcine sludge with these primers (Ballesté et al., 2021). In that microbial source-tracking study, noroviruses did not segregate wastewater of porcine and human origin, contrary to wastewater of bovine and poultry origin. Further genotyping was not conducted in that study. It is beneficial that we have an assay that detects GII NoVs comprehensively, but we need to be aware that while investigating environmental samples, such as surface waters, both PoNoVs and human norovirues, if present in water, may be detected.

### Presence of PoNoVs in Different Age Groups of Pigs

As shown in Table 3, mostly pigs older than 4 months were infected with PoNoVs as compared with younger pigs. There were 17 positive samples (11.8%) from pigs older than 4 months taken on 12 farms, whereas pigs younger than that had only 1 positive sample (2.2%) on one farm.

**Table 3:** Number and percentage of fecal samples positive for porcine noroviruses (PoNoV) in different age groups of pigs

Age groups (months)	Number of PoNoV positive samples/total	% of positive samples
2–4	1/45	2.2
> 4–6	17/144	11.8
Total	18/189	9.5

We focused our study on pigs older than 4 months, since PoNoVs have mostly been detected in pigs of that age in the literature. PoNoVs have frequently been described in finishing pigs (Cavicchio et al., 2020; Nakamura et al., 2010; Scheuer et al., 2013; Silva et al., 2015; Wang et al., 2007). Finding data from literature on PoNoV frequency in pigs less than 4 months of age is challenging. Mijovski et al. (2010) reported a frequency of 1/138 suckling pigs (0.7%). We analyzed a limited number of samples, only one of which contained PoNoV, supporting the general view.

### Genotyping of PoNoVs Positive Samples Using Sanger Sequencing

**Fig. 2:**
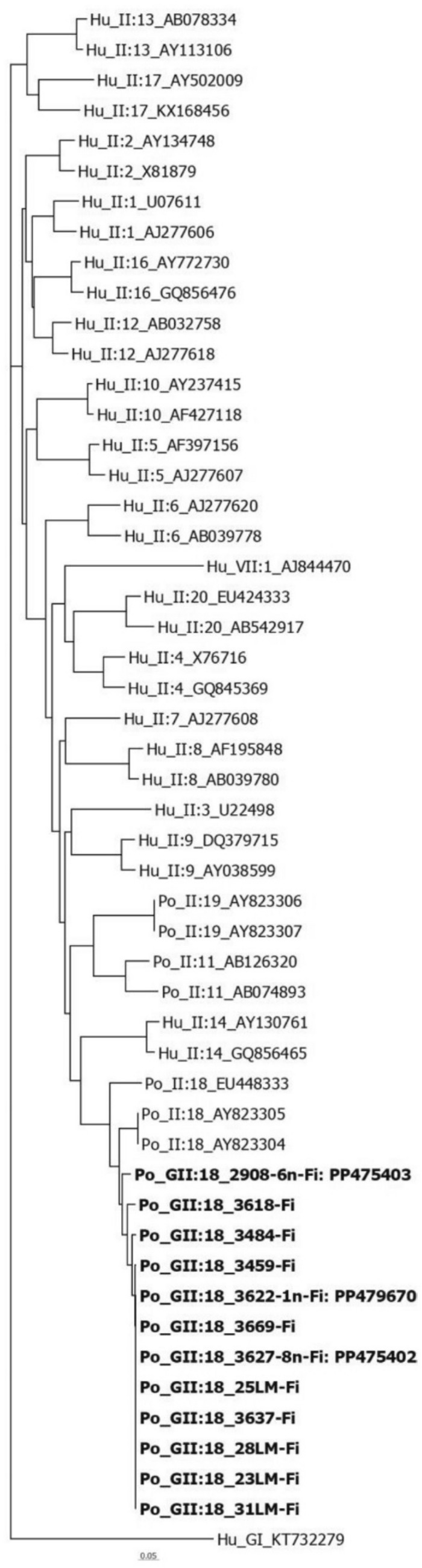
Phylogenetic tree of 12 representative porcine norovirus (PoNoVs) strains obtained in this study and the reference norovirus strains based on 240 bp of the capsid gene region (CLUSTAL OMEGA). The 12 newly identified PoNoV strains are in boldface. Using genogroup I as the outgroup, 20 distinct genotypes from genogroup II are displayed (Po_II:19_AY823306; Po_II:19_AY823307; Po_II:18_AY823305; Po_II:18_AY823304 from USA, Po_II:11_AB126320; Po_II:11_AB074893 from Japan and Po_II:18_EU448333 from Canada.). The strains in the tree were selected based on the tree given by the norovirus genotyping tool. The short sequences do not show the comprehensive evolutionary relationships between the strains

First, we amplified the RT-qPCR-positive samples with the commonly used primer pairs G2SKF/R and p289/290, but only one sample gave a clear band of correct length without background. This partial capsid gene sequence was confirmed as GII.18 using norovirus genotyping tool (3622, GenBank accession no: PP479669). Then, we tested the positive samples with nested RT-PCR targeting the partial capsid gene of GII.18, and 12 of 18 samples gave a band of correct size (240 bp) for PoNoVs. The remaining 6 samples didn’t reveal any bands, and the genotype could not be determined. All 12 strains from this study clustered within GII.18 genotype together with the PoNoV strains QW101 and QW125 (AY823304 and AY823305) from the USA as the closest sequences by BLAST search and by phylogenetic analysis (Fig. 2). Eight positive samples showed 100% nucleic acid identity with each other. Apart from them, one positive sample gave a clearly different GII.18 nucleotide sequence from this cluster (2908, GenBank accession no: PP475403); it shared a pairwise identity of 94–95%. The remaining five had lower quality sequences (some of them had IUPAC; International Union of Pure and Applied Chemistry; nucleotide codes and were not submitted to GenBank). No identical nucleic acid sequences to our sequences of this gene region were available in GenBank. Our PoNoV sequences showed an identity varying from 91 to 92% at nucleotide (nt) level and 100% at amino acid level to the PoNoV QW101 and QW125 strains. The nt identity of our PoNoV strains was 55–56% with the human norovirus GI strain KT732279 (Fig. 2).

After the first GII.11 PoNoV strain (Sw43/1997/JP) was discovered in Japan (Sugieda et al., 1998) the USA and Belgium became the first countries to detect GII.18 and GII.19, respectively (Mauroy et al., 2008; Oka et al., 2013). Since then, according to a recent review by Cavicchio et al. (2022), there have been 47 studies, including those with no evidence of PoNoV, published until the year 2022. All our strains genotyped were assigned to PoNoV GII.18 based on the partial nucleotide sequence of capsid genes. This genotype was also found in the study of Keum et al. (2009), in which they identified three out of four GII.18 PoNoVs and one GII.11 PoNoV based on partial nucleotide sequence of ORF1/ORF2. Wang et al. (2005) reported two positive samples of each genotype GII.11, GII.18, and GII.19. GII.18 strains have also been reported at least in Slovenia, Brazil, Germany, and Italy (Cavicchio et al., 2020; Cunha et al., 2010; Machnowska et al., 2014; Mijovski et al., 2010). As most other studies have used other genome regions for genotype determination than what we used in our study, direct comparison to our sequences is not possible.

We couldn’t not recognize any recombinants in our study, since sequencing of partial polymerase gene was unsuccessful. In future, there is a risk that recombinants between human and porcine noroviruses will evolve. It may be more likely that they evolve among pigs, since human noroviruses are infecting pigs. However, when humans are consuming uncooked oysters contaminated with both viruses, an opportunity for recombination event in human is created.

This study has some limitations. We must interpret our results with caution because only a few samples were collected from each farm. However, our goal was merely to determine whether there were any positive samples; we did not intend to conduct this as a prevalence study. Furthermore, due to the limited number of samples, precise age judgements cannot be made.

This study confirms the presence of GII.18 PoNoVs in Finnish pigs. In future, more research should be focused on recombinants. Also, analysis of a large number of samples might reveal detection of GII.19, GII.11 and possible human norovirus strains in pigs.

## Data Availability

No datasets were generated or analysed during the current study.

## References

[CR1] Atmar, R. L., & Estes, M. K. (2001). Diagnosis of noncultivatable gastroenteritis viruses, the human caliciviruses. *Clinical Microbiology Reviews*. 10.1128/CMR.14.1.15-37.2001 11148001 10.1128/CMR.14.1.15-37.2001 PMC88960

[CR2] Ballesté, E., Blanch, A. R., Mendez, J., Sala-Comorera, L., Maunula, L., Monteiro, S., Farnleitner, A. H., Tiehm, A., Jofre, J., & García-Aljaro, C. (2021). Bacteriophages are good estimators of human viruses present in water. *Frontiers in Microbiology*. 10.3389/fmicb.2021.619495 34012424 10.3389/fmicb.2021.619495 PMC8128106

[CR3] Cavicchio, L., Laconi, A., Piccirillo, A., & Beato, M. S. (2022). Swine norovirus: Past, present, and future. *Viruses*. 10.3390/v14030537 35336944 10.3390/v14030537 PMC8953536

[CR4] Cavicchio, L., Tassoni, L., Laconi, A., Cunial, G., Gagliazzo, L., Milani, A., Campalto, M., Di Martino, G., Forzan, M., Monne, I., & Beato, M. S. (2020). Unrevealed genetic diversity of GII Norovirus in the swine population of North East Italy. *Scientific Reports*. 10.1038/s41598-020-66140-4 32513947 10.1038/s41598-020-66140-4 PMC7280493

[CR5] Chhabra, P., de Graaf, M., Parra, G. I., Chan, M. C. W., Green, K., Martella, V., Wang, Q., White, P. A., Katayama, K., Vennema, H., Koopmans, M. P. G., & Vinjé, J. (2019). Updated classification of norovirus genogroups and genotypes. *Journal of General Virology,**100*(10), 1393–1406. 10.1099/JGV.0.001318 31483239 10.1099/jgv.0.001318 PMC7011714

[CR6] Cunha, J. B., de Mendonça, M. C. L., Miagostovich, M. P., & Leite, J. P. G. (2010). Genetic diversity of porcine enteric caliciviruses in pigs raised in Rio de Janeiro State, Brazil. *Archives of Virology,**155*(8), 1301–1305. 10.1007/s00705-010-0695 20526786 10.1007/s00705-010-0695-z

[CR7] Gyawali, P., Sanjaya, K. C., Beale, D. J., & Hewitt, J. (2019). Current and emerging technologies for the detection of norovirus from shellfish. Foods. 10.3390/foods8060187 10.3390/foods8060187 PMC6617275 31159220

[CR8] Hewitt, J., Rivera-Aban, M., & Greening, G. E. (2009). Evaluation of murine norovirus as a surrogate for human norovirus and hepatitis A virus in heat inactivation studies. *Journal of Applied Microbiology*. 10.1111/j.1365-2672.2009.04179.x 19298511 10.1111/j.1365-2672.2009.04179.x PMC7197740

[CR34] Jiang, X., Huang, P. W., Zhong, W. M., Farkas, T., Cubitt, D. W., & Matson, D. O. (1999). Design and evaluation of a primer pair that detects both Norwalk- and Sapporo-like caliciviruses by RT-PCR. *Journal of Virological Methods*, *83*(1-2), 145–154. 10.1016/s0166-0934(99)00114-7 10.1016/s0166-0934(99)00114-7 10598092

[CR9] Kageyama, T., Kojima, S., Shinohara, M., Uchida, K., Fukushi, S., Hoshino, F. B., Takeda, N., & Katayama, K. (2003). Broadly reactive and highly sensitive assay for Norwalk-like viruses based on real-time quantitative reverse transcription-PCR. *Journal of Clinical Microbiology*. 10.1128/JCM.41.4.1548-1557.2003 12682144 10.1128/JCM.41.4.1548-1557.2003 PMC153860

[CR10] Kantala, T., Oristo, S., Heinonen, M., von Bonsdorff, C. H., & Maunula, L. (2013). A longitudinal study revealing hepatitis E virus infection and transmission at a swine test station. *Research in Veterinary Science*. 10.1016/j.rvsc.2013.09.006 24119762 10.1016/j.rvsc.2013.09.006

[CR11] Kendra, J. A., Tohma, K., & Parra, G. I. (2022). Global and regional circulation trends of norovirus genotypes and recombinants, 1995–2019: A comprehensive review of sequences from public databases. *Reviews in Medical Virology*. 10.1002/rmv.2354 35481689 10.1002/rmv.2354 PMC9542180

[CR12] Keum, H., Moon, H., Park, S., Kim, H., Rho, S., & Park, B. (2009). Porcine noroviruses and sapoviruses on Korean swine farms. *Archives of Virology*. 10.1007/s00705-009-0501-y 19812890 10.1007/s00705-009-0501-y

[CR35] Kojima, S., Kageyama, T., Fukushi, S., Hoshino, F. B., Shinohara, M., Uchida, K., Natori, K., Takeda, N., & Katayama, K. (2002). Genogroup-specific PCR primers for detection of Norwalk-like viruses. *Journal of virological methods.**100*(1-2), 107–114. 10.1016/s0166-0934(01)00404-9 10.1016/s0166-0934(01)00404-9 11742657

[CR13] L’Homme, Y., Sansregret, R., & Simard, C. (2009). Broad range RT-PCR assays targeting human noroviruses also detect swine noroviruses. *Food Microbiology*. 10.1016/j.fm.2009.03.011 19465254 10.1016/j.fm.2009.03.011

[CR14] Lefkowitz, E. J., Dempsey, D. M., Hendrickson, R. C., Orton, R. J., Siddell, S. G., & Smith, D. B. (2018). Virus taxonomy: The database of the International Committee on Taxonomy of Viruses (ICTV). *Nucleic Acids Research*. 10.1093/nar/gkx932 29040670 10.1093/nar/gkx932 PMC5753373

[CR15] Loisy, F., Atmar, R. L., Guillon, P., le Cann, P., Pommepuy, M., & le Guyader, F. S. (2005). Real-time RT-PCR for norovirus screening in shellfish. *Journal of Virological Methods*. 10.1016/j.jviromet.2004.08.023 15582692 10.1016/j.jviromet.2004.08.023

[CR16] Machnowska, P., Ellerbroek, L., & Johne, R. (2014). Detection and characterization of potentially zoonotic viruses in faeces of pigs at slaughter in Germany. *Veterinary Microbiology*. 10.1016/j.vetmic.2013.10.018 24247020 10.1016/j.vetmic.2013.10.018

[CR17] Mattison, K., Grudeski, E., Auk, B., Brassard, J., Charest, H., Dust, K., Gubbay, J., Hatchette, T. F., Houde, A., Jean, J., Jones, T., Lee, B. E., Mamiya, H., McDonald, R., Mykytczuk, O., Pang, X., Petrich, A., Plante, D., Ritchie, G., … Booth, T. F. (2011). Analytical performance of norovirus real-time RT-PCR detection protocols in Canadian laboratories. *Journal of Clinical Virology*, *50*(2), 109–113. 10.1016/j.jcv.2010.10.008 10.1016/j.jcv.2010.10.008 21071266

[CR18] Mattison, K., Shukla, A., Cook, A., Pollari, F., Friendship, R., Kelton, D., Bidawid, S., & Farber, J. M. (2007). Human noroviruses in swine and cattle. *Emerging Infectious Diseases*. 10.3201/eid1308.070005 17953089 10.3201/eid1308.070005 PMC2828081

[CR19] Mauroy, A., Scipioni, A., Mathijs, E., Miry, C., Ziant, D., Thys, C., & Thiry, E. (2008). Noroviruses and sapoviruses in pigs in Belgium. *Archives of Virology,**153*(10), 1927–1931. 10.1007/s00705-008-0189-4 18777158 10.1007/s00705-008-0189-4

[CR20] Mijovski, J. Z., Poljšak-Prijatelj, M., Steyer, A., Barlič-Maganja, D., & Koren, S. (2010). Detection and molecular characterisation of noroviruses and sapoviruses in asymptomatic swine and cattle in Slovenian farms. *Infection, Genetics and Evolution,**10*(3), 413–420. 10.1016/j.meegid.2009.11.010 19931649 10.1016/j.meegid.2009.11.010

[CR21] Nakamura, K., Saga, Y., Iwai, M., Obara, M., Horimoto, E., Hasegawa, S., Kurata, T., Okumura, H., Nagoshi, M., & Takizawa, T. (2010). Frequent detection of noroviruses and sapoviruses in swine and high genetic diversity of porcine sapovirus in Japan during fiscal year 2008. *Journal of Clinical Microbiology*. 10.1128/JCM.02130-09 20164276 10.1128/JCM.02130-09 PMC2849587

[CR22] Oka, T., Saif, L. J., & Wang, Q. (2013). First complete genome sequence of a genogroup II genotype 18 porcine norovirus, strain QW125. *Genome Announcements*. 10.1128/genomeA.00344-13 23766405 10.1128/genomeA.00344-13 PMC3707576

[CR36] Parra G. I. (2019). Emergence of norovirus strains: A tale of two genes. *Virus Evolution*, *5*(2), vez048. 10.1093/ve/vez048 10.1093/ve/vez048 PMC6875644 32161666

[CR23] Pintó, R. M., Costafreda, M. I., & Bosch, A. (2009). Risk assessment in shellfish-borne outbreaks of hepatitis A. *Applied and Environmental Microbiology,**75*(23), 7350–7355. 10.1128/AEM.01177-09 19820160 10.1128/AEM.01177-09 PMC2786421

[CR24] Robilotti, E., Deresinski, S., & Pinsky, B. A. (2015). Norovirus. *Clinical Microbiology Reviews,**28*(1), 134–164. 10.1128/CMR.00075-14 25567225 10.1128/CMR.00075-14 PMC4284304

[CR25] Scheuer, K. A., Oka, T., Hoet, A. E., Gebreyes, W. A., Molla, B. Z., Saif, L. J., & Wang, Q. (2013). Prevalence of porcine Noroviruses, molecular characterization of emerging porcine sapoviruses from finisher swine in the United States, and unified classification scheme for sapoviruses. *Journal of Clinical Microbiology*. 10.1128/JCM.00865-13 23678065 10.1128/JCM.00865-13 PMC3697660

[CR39] Schuele, L., Lizarazo-Forero, E., Strutzberg-Minder, K., Schütze, S., Löbert, S., Lambrecht, C., Harlizius, J., Friedrich, A. W., Peter, S., Rossen, J. W. A., & Couto, N. (2022). Application of shotgun metagenomics sequencing and targeted sequence capture to detect circulating porcine viruses in the Dutch-German border region. *Transboundary and Emerging Diseases,**69*(4), 2306–2319. 10.1111/tbed.14249 10.1111/tbed.14249 PMC9540031 34347385

[CR37] Shen, Q., Zhang, W., Yang, S., Yang, Z., Chen, Y., Cui, L., Zhu, J., & Hua, X. (2012). Recombinant porcine norovirus identified from piglet with diarrhea. *BMC Veterinary Research,**8*, 155. 10.1186/1746-6148-8-155 10.1186/1746-6148-8-155 PMC3514297 22938017

[CR26] Silva, P. F. N., Alfieri, A. F., Barry, A. F., de Arruda Leme, R., Gardinali, N. R., van der Poel, W. H. M., & Alfieri, A. A. (2015). High frequency of porcine norovirus infection in finisher units of Brazilian pig-production systems. *Tropical Animal Health and Production,**47*(1), 237–241. 10.1007/s11250-014-0685-3 25281212 10.1007/s11250-014-0685-3

[CR27] Sugieda, M., Nagaoka, H., Kakishima, Y., Ohshita, T., Nakamura, S., & Nakajima, S. (1998). Detection of Norwalk-like virus genes in the caecum contents of pigs. *Brief Report. Archives of Virology*. 10.1007/s007050050369 10.1007/s007050050369 9687878

[CR28] Summa, M., von Bonsdorff, C. H., & Maunula, L. (2012). Evaluation of four virus recovery methods for detecting noroviruses on fresh lettuce, sliced ham, and frozen raspberries. *Journal of Virological Methods,**183*(2), 154–160. 10.1016/j.jviromet.2012.04.006 22580195 10.1016/j.jviromet.2012.04.006

[CR29] van der Poel, W. H. M., Vinjé, J., van der Heide, R., Herrera, M. I., Vivo, A., & Koopmans, M. P. G. (2000). Norwalk-like calicivirus genes in farm animals. *Emerging Infectious Diseases*. 10.3201/eid0601.000106 10653567 10.3201/eid0601.000106 PMC2627973

[CR30] Wang, Q. H., Costantini, V., & Saif, L. J. (2007). Porcine enteric caliciviruses: Genetic and antigenic relatedness to human caliciviruses, diagnosis and epidemiology. *Vaccine,**25*(30 Spec. ISS), 5453–5466. 10.1016/j.vaccine.2006.12.032 17234307 10.1016/j.vaccine.2006.12.032 PMC2735111

[CR31] Wang, Q. H., Myung, G. H., Cheetham, S., Souza, M., Funk, J. A., & Saif, L. J. (2005). Porcine noroviruses related to human noroviruses. *Emerging Infectious Diseases*. 10.3201/eid1112.050485 16485473 10.3201/eid1112.050485 PMC3367634

[CR32] Wang, Q. H., Souza, M., Funk, J. A., Zhang, W., & Saif, L. J. (2006). Prevalence of noroviruses and sapoviruses in swine of various ages determined by reverse transcription-PCR and microwell hybridization assays. *Journal of Clinical Microbiology*. 10.1128/JCM.02634-05 16757598 10.1128/JCM.02634-05 PMC1489419

[CR33] Wolf, S., Williamson, W., Hewitt, J., Lin, S., Rivera-Aban, M., Ball, A., Scholes, P., Savill, M., & Greening, G. E. (2009). Molecular detection of norovirus in sheep and pigs in New Zealand farms. *Veterinary Microbiology*. 10.1016/j.vetmic.2008.06.019 18676104 10.1016/j.vetmic.2008.06.019

[CR38] Sisay, Z., Djikeng, A., Berhe, N., Belay, G., Abegaz, W. E., Wang, Q. H., & Saif, L. J. (2016). First detection and molecular characterization of sapoviruses and noroviruses with zoonotic potential in swine in Ethiopia. *Archives of Virology,**161*(10), 2739–2747. 10.1007/s00705-016-2974-9 10.1007/s00705-016-2974-9 27424025

